# ﻿Two new species of *Stilbochaeta* (Chaetosphaeriaceae, Chaetosphaeriales) from freshwater and terrestrial habitats in China

**DOI:** 10.3897/mycokeys.121.158160

**Published:** 2025-08-21

**Authors:** Wangming Zhang, Qinying Feng, Xiaoyu Song, Xinzhong Zhou, Wanqing Xie, Juan Lu, Li Lu

**Affiliations:** 1 Beijing Jishuitan Hospital Guizhou Hospital, Guiyang, China Beijing Jishuitan Hospital Guizhou Hospital Guiyang China

**Keywords:** Asexual morph, phylogeny, Sordariomycetes, taxonomy, two new species

## Abstract

During a taxonomic investigation of saprobic fungi in the tropical and subtropical regions of southern China, four hyphomycetous isolates were obtained from freshwater and terrestrial substrates. Phylogenetic analyses, based on combined LSU and ITS rDNA sequence data, coupled with detailed morphological comparisons, revealed that two of the isolates represent previously undescribed species within the genus *Stilbochaeta*. The new taxa, *Stilbochaeta guizhouensis* and *S. hainanensis*, are introduced herein with comprehensive descriptions and illustrations. The present study expands the known diversity of *Stilbochaeta* and enhances the understanding of asexual fungal taxa in both aquatic and terrestrial ecosystems in China.

## ﻿Introduction

*Stilbochaeta* was established by Réblová et al. (2021) with *S. malaysiana* designated as the type species, based on both morphological characteristics and phylogenetic evidence. In their study, eight species were introduced: *Stilbochaeta aquatica*, *S. brevisetula*, *S. cangshanensis*, *S. malaysiana*, *S. novae-guineensis*, *S. ramulosetula*, *S. septata* and *S. submersa* (Hughes and Kendrick 1968; Matsushima 1971; Romero 1983; Kuthubutheen 1987a, 1987b; Whitton et al. 2000; Wei et al. 2018; Luo et al. 2019; Hsieh et al. 2021; Réblová et al. 2021). Subsequently, Wu and Diao (2022) conducted a study on anamorphic chaetosphaeriaceous fungi within the family Chaetosphaeriaceae, incorporating both DNA sequence data and morphological characteristics. They introduced three additional species: *S. ejneri*, *S. minteri* and *S. sinensis* and proposed a new combination *S. jianfenglingensis* (Xia et al. 2015; Wu and Diao 2022).

To date, *Stilbochaeta* contains 12 species, based on morphological and/or molecular data, with only *S. jianfenglingensis* lacking molecular data (Xia et al. 2015; Réblová et al. 2021; Wu and Diao 2022). *Stilbochaeta* species are distributed in Australia, Brazil, Chile, China, Cuba, Ivory Coast, Malaysia, New Zealand, Philippines, Puerto Rico, Solomon Islands, South Africa, Thailand and the Caribbean (Wei et al. 2018; Luo et al. 2019; Hsieh et al. 2021; Réblová et al. 2021; Wu and Diao 2022). They occur as saprobes on leaf litter, bamboo and decaying bark, branch, fruit, leaves, stem and wood in both freshwater and terrestrial habitats (Luo et al. 2019; Hsieh et al. 2021; Réblová et al. 2021; Wu and Diao 2022). Amongst the reported *Stilbochaeta* species, 11 are known only from their asexual morphs, while only *S. brevisetula* exhibits both asexual and sexual morphs (Xia et al. 2015; Wei et al. 2018; Luo et al. 2019; Hsieh et al. 2021; Réblová et al. 2021; Wu and Diao 2022). The asexual morph of *Stilbochaeta* is characterised by erect, straight or flexuous, septate, unbranched, sterile setae; hairy, brown, colonies with white, glistening conidial mass; macronematous, erect, straight or bent, septate conidiophores; integrated, terminal, mono- or polyphialidic, subcylindrical, pale brown conidiogenous cells with funnel-shaped collarettes, and septate, hyaline conidia (Luo et al. 2019; Hsieh et al. 2021; Réblová et al. 2021; Wu and Diao 2022). The sexual morph is characterised by globose to subglobose, papillate, dark brown ascomata with a periphysate ostiole; cylindrical to clavate, stipitate asci with rounded apices; and fusiform, hyaline, septate ascospores (Hughes and Kendrick 1968; Whitton et al. 2000; Réblová et al. 2021).

In the present study, four hyphomycetous fungal isolates representing two distinct taxa were obtained from both freshwater and terrestrial habitats in Guizhou and Hainan Provinces, China. Detailed morphological characterisation, comprehensive illustrations and molecular phylogenetic analyses support the recognition of these isolates as two novel species within the genus *Stilbochaeta*. Accordingly, we propose *Stilbochaeta guizhouensis* sp. nov. and *S. hainanensis* sp. nov. as new taxa.

## ﻿Materials and methods

### ﻿Sample collection and specimen examination

Fresh specimens were collected from freshwater and terrestrial habitats in Qianxinan Buyi and Miao Autonomous Prefecture, Guizhou Province and Wuzhishan City, Hainan Province, China. Samples were taken to the laboratory in plastic bags, labelled with collection details, including locality, habitat and date (Rathnayaka et al. 2024). Specimens from freshwater habitats were cultured at room temperature and maintained in a moist environment for about two weeks. The samples were examined using a stereomicroscope (SMZ 745, Nikon, Japan). Micro-morphological characters were captured using a Nikon EOS 90D digital camera attached to an ECLIPSE Ni compound microscope (Nikon, Japan). Measurements of conidiophores, conidiogenous cells and conidia were carried out using the Tarosoft (R) Image Frame Work programme.

### ﻿Isolation and material deposition

Single-spore isolation was performed following the method described by Senanayake et al. (2020). The germinated conidia were aseptically transferred to fresh potato dextrose agar (PDA) and incubated at room temperature for 36–44 days. Morphological characteristics of the fungal mycelium on PDA, including colour, shape, size, margin and elevation, were documented. Dried fungal specimens were deposited in the Herbarium of Guizhou Academy of Agriculture Sciences (Herb. GZAAS), Guiyang, China. Pure cultures were deposited at the Guizhou Culture Collection (GZCC), Guiyang, China. Descriptions of the new taxa were uploaded to the Faces of Fungi webpage following the guidelines of Jayasiri et al. (2015). The new species were registered in the MycoBank database (https://www.mycobank.org/) and MycoBank numbers were obtained.

### ﻿DNA extraction, PCR amplification and sequencing

Fresh fungal mycelia grown on PDA were scraped using sterilised scalpels. Genomic DNA was extracted using the Biospin Fungus Genomic DNA Extraction Kit (BioFlux, China), following the manufacturer’s protocol. The primer pairs ITS5/ITS4 (White et al. 1990) and LR0R/LR5 (Vilgalys and Hester 1990) were used to amplify the ITS and LSU regions, respectively. PCR amplification was performed in a 25 μl reaction volume, consisting of 13.5 μl of 10 × PCR Master Mix, 1 μl of each primer, 1 μl template DNA and 8.5 μl ddH_2_O. The thermocycling conditions were as follows: initial denaturation at 94 °C for 3 min; 40 cycles of denaturation at 94 °C for 45 s, annealing at 56 °C for 50 s and extension at 72 °C for 1 min; followed by a final extension at 72 °C for 10 min. The PCR products were purified and sequenced by Sangon Biotech (Shanghai, China) Co., Ltd.

### ﻿Phylogenetic analyses

BioEdit v. 7.0.5.3 (Hall 1999) and SeqMan v. 7.0.0 (Swindell and Plasterer 1997) were used to check and assemble the newly-generated sequences. Sequences obtained in this study (Table 1) were downloaded from the NCBI GenBank database (https://blast.ncbi.nlm.nih.gov/Blast.cgi). Multiple sequence alignments for each locus dataset were performed using MAFFT v.7.473 (https://mafft.cbrc.jp/alignment/server/, Katoh et al. (2019) and visually inspected in AliView (Larsson 2014). The LSU and ITS alignments were trimmed using trimAl v.1.2rev59 (Capella-Gutiérrez et al. 2009) and subsequently merged using SequenceMatrix v.1.7.8 (Vaidya et al. 2011).

**Table 1.** Taxa incorporated in this study and their respective GenBank accession numbers.

Taxon	Strain	GenBank Accession numbers		References
		LSU	ITS	
*Codinaea assamica*	CBS 139907^T^	OL654134	OL654077	Réblová et al. (2021)
*Codinaea dwaya*	CBS 261.77^T^	OL654135	OL654078	Réblová et al. (2021)
*Falholtia kaohsiungensis*	NN050711	OL655083	OL627699	Hsieh et al. (2021); Wu and Diao (2022)
*Falholtia kaohsiungensis*	BCRC FU31337^T^	NG_075392	NR_172187	Hsieh et al. (2021)
*Stilbochaeta aquatica*	NN076652	OL655190	OL628137	Réblová et al. (2021)
*Stilbochaeta aquatica*	MFLU 15-2691^T^	MH476569	MH476572	Wei et al. (2018); Réblová et al. (2021)
*Stilbochaeta aquatica*	CBS 114070	OL654172	OL654115	Réblová et al. (2021)
*Stilbochaeta brevisetula*	ICMP 22549^E^	OL654175	OL654118	Réblová et al. (2021)
*Stilbochaeta cangshanensis*	NN047608	OL655063	OL627667	Wu and Diao (2022)
*Stilbochaeta cangshanensis*	NN077955	OL655247	OL628479	Wu and Diao (2022)
*Stilbochaeta cangshanensis*	MFLU 18-1614^T^	MK835832	MK828632	Luo et al. (2019); Wu and Diao (2022)
*Stilbochaeta ejneri*	CGMCC 3.20716^T^	OL655138	OL627877	Wu and Diao (2022)
***Stilbochaeta guizhouensis***	GZCC 25-0001^T^	PV771042	PV771038	This study
***Stilbochaeta guizhouensis***	GZCC 25-0002	PV771043	PV771039	This study
***Stilbochaeta hainanensis***	GZCC 25-0003^T^	PV771044	PV771040	This study
***Stilbochaeta hainanensis***	GZCC 25-0004	PV771045	PV771041	This study
*Stilbochaeta malaysiana*	NN076748	OL655198	OL628162	Wu and Diao (2022)
*Stilbochaeta malaysiana*	NN076617	OL655187	OL628130	Wu and Diao (2022)
*Stilbochaeta malaysiana*	IMI 312436^T^	OL654178	OL654121	Réblová et al. (2021)
*Stilbochaeta minteri*	NN076642	OL655188	OL628135	Wu and Diao (2022)
*Stilbochaeta novae-guineensis*	CBS 147515	OL654179	OL654122	Réblová et al. (2021)
*Stilbochaeta ramulosetula*	IMI 313452^E^	OL654181	OL654124	Réblová et al. (2021)
*Stilbochaeta septata*	CBS 143386^E^	MH107936	MH107889	Réblová et al. (2021)
*Stilbochaeta septata*	CBS 146716	OL654182	OL654125	Réblová et al. 2021)
*Stilbochaeta sinensis*	NN076599	OL655182	OL628119	Wu and Diao (2022)
*Stilbochaeta sinensis*	NN076325	OL655168	OL628070	Wu and Diao (2022)
*Stilbochaeta sinensis*	NN055280	OL655123	OL627810	Wu and Diao (2022)
*Stilbochaeta sinensis*	NN054248	OL655099	OL627741	Wu and Diao (2022)
*Stilbochaeta submersa*	NN047996	OL655072	OL627682	Wu and Diao (2022)
*Stilbochaeta submersa*	MFLU 18-2321^T^	MK835831	MK828631	Luo et al. (2019); Réblová et al. (2021)

Note: The newly-obtained strains are in bold red. “^T^” and “^E^” represent ex-type and ex-epitype strains.

Maximum Likelihood (ML) analysis was conducted using the IQ-TREE web server (http://iqtree.cibiv.univie.ac.at/), based on Bayesian Information Criteria (BIC) (Nguyen et al. 2015). The substitution model was automatically selected by the server. Bayesian Inference (BI) analysis was performed using MrBayes on XSEDE (3.2.7a) via the CIPRES Science Gateway (Stamatakis 2014). The aligned FASTA file was converted to NEXUS format using AliView (Daniel et al. 2010). The best-fit evolutionary model for each dataset was determined using MrModelTest v. 2.3. 10 (Nylander 2004). The GTR+I+G substitution model was selected for both the ITS and LSU regions. The posterior probabilities (PP) were determined, based on Bayesian Markov Chain Monte Carlo (BMCMC) sampling (Huelsenbeck and Ronquist 2001). Two simultaneous Markov chains were run for 10,000,000 generations and trees sampled every 1,000^th^ generation. The burn-in phase was set at 25% and the remaining trees were used to calculate posterior probabilities (BYPP).

Phylogenetic trees were visualised using FigTree v. 1.4.4 and further edited in PowerPoint. The photo-plate was made using Adobe Photoshop CS6 software (Adobe Systems, the USA).

### ﻿Phylogenetic Results

**Figure 1.**
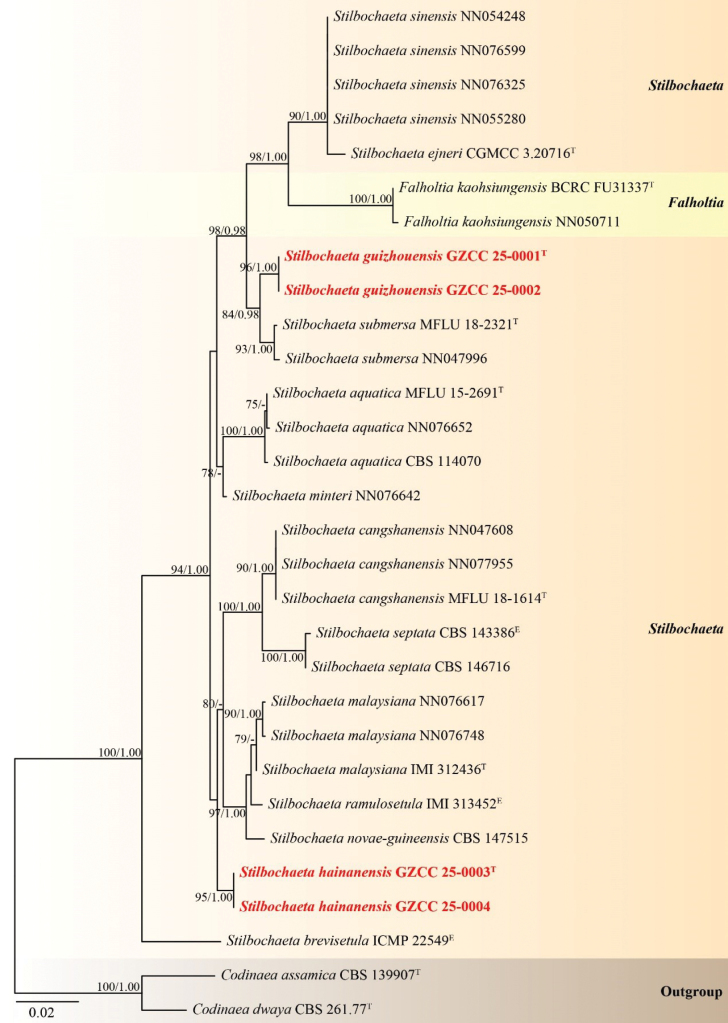
Phylogenetic tree generated from the RAxML analysis, based on the combined dataset of LSU and ITS sequences. Bootstrap support values for ML (≥ 75%) and BYPP (≥ 0.95) are indicated near their respective nodes. *Codinaea assamica* (CBS 139907) and *C. dwaya* (CBS 261.77) were selected as outgroup taxa. Ex-type and ex-epitype strains are denoted by “^T^” and “^E^”, respectively and newly-obtained isolates are indicated in bold red font. A dash (“-”) indicates bootstrap support values below 0.95 for BYPP.

Partial nucleotide sequences of the ITS and LSU regions were used to determine the phylogenetic position of our newly-obtained strains. The Maximum Likelihood and Bayesian Inference analyses yielded similar tree topologies. A total of 24 strains, including four newly-isolated strains and two outgroup taxa, *Codinaea assamica* (CBS 139907) and *C. dwaya* (CBS 261.77), were included in the analysis. The concatenated sequence matrix consisted of 1,344 characters (ITS = 501 bp and LSU = 843 bp). Fig. 1 illustrates the best-scoring RAxML tree, with a final likelihood value of -3579.590.

In the phylogenetic tree (Fig. 1), our collections represent two distinct novel *Stilbochaeta* species within Chaetosphaeriaceae. Our isolates, GZCC 25–0001 and GZCC 25–0002 group together and are sister to *Stilbochaeta submersa* (MFLU 18–2321 and NN047996), supported by 84% ML and 0.98 BYPP. Additionally, our isolates (GZCC 25–0003 and GZCC 25–0004) clustered together, forming a distinct lineage with *S. cangshanensis*, *S. malaysiana*, *S. novae-guineensis*, *S. ramulosetula* and *S. septata*, albeit with low statistical support.

## ﻿Taxonomy

### ﻿ Stilbochaeta guizhouensis

Taxon classificationFungiChaetosphaerialesChaetosphaeriaceae

W.M. Zhang & L. Lu sp. nov.

AE1AA39F-98DC-5F13-98F9-269F9A89305C

572743

Facesoffungi Number: FoF17800

Fig. 2

#### Etymology.

The epithet “*guizhouensis*” refers to Guizhou Province, China, where the fungus was collected.

#### Holotype.

GZAAS 25–0001.

#### Description.

***Saprobic*** on decaying wood in a terrestrial habitat. ***Sexual morph*** Undetermined. ***Asexual morph*** Hyphomycetous. ***Colonies*** on the natural substrate effuse, hairy, brown, with white, glistening conidial mass. ***Mycelium*** mostly immersed, composed of branched, septate, smooth, brown hyphae. ***Setae*** 170–251 µm long, 5–7 µm wide at the base (x̄ = 210 × 6 µm, n = 15), erect, slightly curved, dark brown at the base, pale brown towards the apex, apical cell with a round end, septate, unbranched, smooth. ***Conidiophores*** 63–251 µm long, 4–7 µm wide at the base (x̄ = 122 × 5.5 µm, n = 15), macronematous, mononematous, brown at the base, fading to pale brown towards the apex, septate, unbranched, cylindrical, erect, straight or slightly curved, thin-walled, smooth, arising in groups from the bases of setae. ***Conidiogenous cells*** 19–30.5 µm long, 3.5–5 µm wide (x̄ = 23.5 × 4.5 µm, n = 25), mono- or poly-phialidic integrated, terminal, determinate, clavate, ellipsoidal, collarette funnel-shaped. ***Conidia*** 10–16 µm long, 2–3.5 µm wide (x̄ = 14.5 × 3 µm, n = 20), acrogenous, usually aggregated in slimy droplets, hyaline, 1-septate, guttules, oblong to allantoid, slightly curved, rounded at ends, smooth, with 5–12.5 μm long hair–like appendages at both ends.

**Figure 2.**
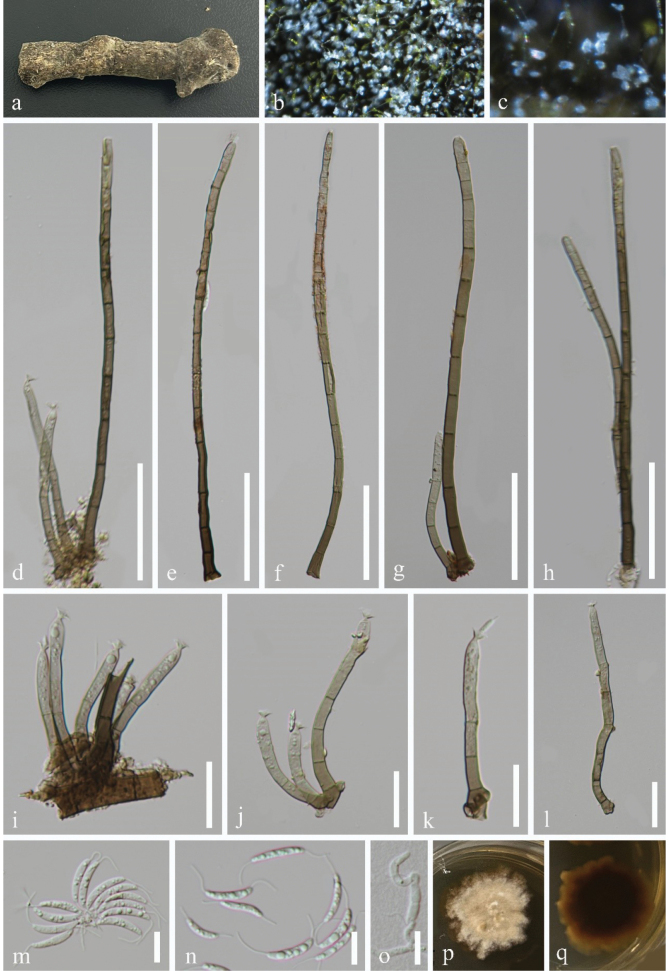
*Stilbochaeta guizhouensis* (GZAAS 25–0001, holotype). a–c. Colonies on the natural substrate; d–h. Setae and conidiophores; i–l. Conidiophores and conidiogenous cells bearing funnel-shaped collarettes; m, n. Conidia; o. Germinated conidium; p, q Colonies on PDA from above and below. Scale bars: 50 μm (d–h); 20 μm (i–l); 10 μm (m–o).

#### Culture characteristics.

Conidia germinating on PDA within 9 h and germ tubes arising from the terminal ends of the conidium. Colonies reached 32 mm diam. after 36 days of incubation at 25 °C, irregular, with flat, pale brown to brown mycelia on the surface, in reverse, brown to black-brown with undulate margin.

#### Material examined.

China, Guizhou Province, Qianxinan Buyei and Miao Autonomous Prefecture, on decaying wood in a terrestrial habitat, 28 November 2024, Wang-Ming Zhang, GC16 (GZAAS 25–0001, holotype), ex-type living culture GZCC 25–0001; *Ibid*., GC18 (GZAAS 25–0002, paratype), living culture GZCC 25–0002.

#### Notes.

In the phylogenetic tree (Fig. 1), our isolates GZCC 25–0001 and GZCC 25–0002 clustered together with 100% ML and 1.00 BYPP support, indicating that they belong to the same species. This clade formed a distinct lineage with *Stilbochaeta submersa* (MFLU 18–2321 and NN047996), supported by 84% ML and 0.98 BYPP, indicating that they represent two different species. A comparison of ITS and LSU sequences between GZCC 25–0001 and MFLU 18–2321 revealed nucleotide base differences of 11/533 bp (2.1%, including one gap) and 5/797 bp (0.6%, without a gap), respectively. Morphologically, *Stilbochaeta guizhouensis* (GZAAS 25–0001) can be distinguished from *Stilbochaeta submersa* (MFLU 18–2321) by longer conidiophores (up to 251 µm long vs. 62–122(–152) µm long) and larger conidiogenous cells (19–30.5 µm long, 3.5–5 µm wide vs. 13.5–16.5 µm long, 2.5–3.5 µm wide; Luo et al. (2019); Wu and Diao (2022)). Therefore, based on the findings from both molecular and morphological evidence (Chethana et al. 2021), we introduce the two isolates, GZCC 25–0001 and GZCC 25–0002, as a new species, namely *Stilbochaeta guizhouensis*.

### ﻿ Stilbochaeta hainanensis

Taxon classificationFungiChaetosphaerialesChaetosphaeriaceae

W.M. Zhang & L. Lu sp. nov.

1FB49739-0195-5B6C-8DC7-160D53477310

572744

Facesoffungi Number: FoF17801

Fig. 3

#### Etymology.

The epithet “*hainanensis*” refers to Hainan Province, China, where the fungus was collected.

#### Holotype.

GZAAS 25–0003.

#### Description.

***Saprobic*** on submerged decaying wood in a freshwater habitat. ***Sexual morph*** Undetermined. ***Asexual morph*** Hyphomycetous. ***Colonies*** on the natural substrate effuse, hairy, brown, with white, glistening conidial mass. ***Mycelium*** mostly immersed, composed of branched, septate, smooth, brown hyphae. ***Conidiophores*** 70–171 µm long, 3.5–6 µm wide at the base (x̄ = 125.5 × 5 µm, n = 20), macronematous, mononematous, brown at the base, fading to pale brown towards the apex, septate, unbranched, cylindrical, erect, straight or slightly curved, thin-walled, smooth. ***Conidiogenous cells*** 11–26 µm long, 3–5.5 µm wide (x̄ = 21 × 4.5 µm, n = 25), mono- or poly-phialidic integrated, terminal, determinate, clavate, ellipsoidal, collarette funnel-shaped. ***Conidia*** 8.5–14.5 µm long, 2.5–4 µm wide (x̄ = 10.5 × 3.5 µm, n = 30), acrogenous, usually aggregated in slimy droplets, 1-septate, guttules, subcylindrical, obclavate, fusiform to allantoid, slightly curved, hyaline.

**Figure 3.**
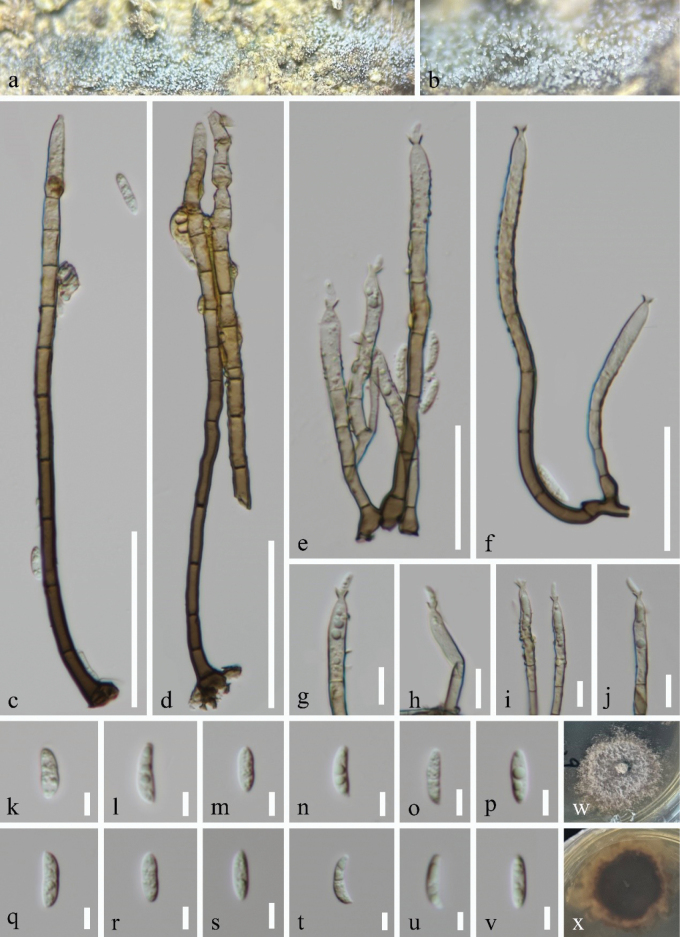
*Stilbochaeta hainanensis* (GZAAS 25–0003, holotype). a, b. Colonies on the natural substrate; c–f. Conidiophores and conidiogenous cells bearing funnel-shaped collarettes; g–j. Conidiogenous cells; k–v. Conidia; w, x. Colonies on PDA from above and below. Scale bars: 50 μm (c, d); 30 μm (e, f); 10 μm (g–j); 5 μm (k–v).

#### Culture characteristics.

Conidia germinating on PDA within 11 h and germ tubes arising from the conidium. Colonies reached 38 mm diam. after 44 days of incubation at 25 °C, irregular, with flat, white to pale brown mycelia on the surface, in reverse pale brown to brown, with undulate margin.

#### Material examined.

China, Hainan Province, Wuzhishan City, on submerged decaying wood in a freshwater habitat, 10 January 2025, Wang-Ming Zhang, W25 (GZAAS 25–0003, holotype), ex-type living culture GZCC 25–0003; *Ibid*., W27.3 (GZAAS 25–0004, paratype), living culture GZCC 25–0004.

#### Notes.

*Stilbochaeta hainanensis* aligns with the asexual morphological species concept of the genus *Stilbochaeta*, as defined by Réblová et al. (2021) and Wu and Diao (2022). In our phylogenetic analysis (Fig. 1), *Stilbochaeta hainanensis* (GZCC 25–0003 and GZCC 25–0004) clusters with *S. cangshanensis* (MFLUCC 17–2214, NN047608 and NN077955), *S. malaysiana* (IMI 312436, NN076617 and NN076748), *S. novae-guineensis* (CBS 147515), *S. ramulosetula* (IMI 313452) and *S. septata* (CBS 143386 and CBS 146716). However, *Stilbochaeta hainanensis* (8.5–14.5 µm long conidia) differs from *S. cangshanensis* (15–18 µm long), *S. malaysiana* (21–26 µm long), *S. novae-guineensis* (14–19 µm long), *S. ramulosetula* (16–19.5 µm long) and *S. septata* (13.5–16 µm long) by having smaller conidia (Réblová et al. 2021). In addition, *Stilbochaeta hainanensis* (70–171 µm long) can be distinguished from *S. cangshanensis* (39–53 µm long) and *S. novae-guineensis* (27.5–58 µm long) by its longer conidiophores (Réblová et al. 2021). Furthermore, our isolates GZCC 25–0003 and GZCC 25–0004 form a strongly-supported monophyletic group with 100% ML and 1.00 BYPP values, indicating they represent the same species. Within *Stilbochaeta*, this lineage is distinct from all previously known taxa. Therefore, based on multi-gene phylogenetic analyses, we introduce GZCC 25–0003 and GZCC 25–0004 as representing a new species, *Stilbochaeta hainanensis*.

## ﻿Discussion

In this study, the genus *Stilbochaeta* currently comprises 14 recognised species, including the newly-described *S. guizhouensis* and *S. hainanensis* (Hsieh et al. 2021; Réblová et al. 2021; Wu and Diao 2022). Amongst these, 10 species have been reported from freshwater and terrestrial habitats in China, suggesting a regional concentration of diversity. This phenomenon may be attributed to the limited geographical scope of sampling collection.

Previous molecular and multi-gene phylogenetic analyses have shown that the three asexual genera *Codinaea*, *Dictyochaeta* and *Stilbochaeta* within the Chaetosphaeriaceae share similar morphological features. These include clustered conidiophores associated with setae, terminal mono- or polyphialidic conidiogenous cells with funnel-shaped collarettes and hyaline, septate conidia (Hsieh et al. 2021; Réblová et al. 2021; Zhang et al. 2022; Wu and Diao 2022). Accurate identification of species within *Codinaea*, *Dictyochaeta* and *Stilbochaeta*, based solely on morphological characteristics, remains challenging (Hsieh et al. 2021; Réblová et al. 2021; Zhang et al. 2022; Wu and Diao 2022). Therefore, molecular data should be integrated with subtle morphological characteristics to achieve accurate identification and classification of these taxa.

The relationship between *Falholtia kaohsiungensis* and *Stilbochaeta* species is noteworthy based on molecular evidence. For example, *Falholtia kaohsiungensis* (BCRC FU31337 and NN050711) forms a sister clade to *Stilbochaeta ejneri* (CGMCC 3.20716) and *S. sinensis* (NN054248, NN055280, NN076325 and NN076599), supported by 100% ML and 1.00 BYPP, indicating that they belong to the same genus. However, *Falholtia kaohsiungensis* differs morphologically from the asexual morph of *Stilbochaeta* by possessing cylindrical and dark brown conidiophores in clusters or synnemata, monoblastic conidiogenous cells, brown, obclavate-rostrate, euseptate conidia (Lin et al. 2019; Luo et al. 2019; Hsieh et al. 2021; Réblová et al. 2021; Wu and Diao 2022; Zhang et al. 2022). Therefore, accurate taxonomic classification of these taxa requires the integration of molecular and morphological data. This study enriches our understanding of fungal diversity in tropical and subtropical ecosystems and underscores the importance of combining molecular tools with traditional taxonomy in fungal systematics.

## Supplementary Material

XML Treatment for Stilbochaeta guizhouensis

XML Treatment for Stilbochaeta hainanensis

## ﻿Additional information

### Use of AI

No use of AI was reported.

### Data availability

All of the data that support the findings of this study are available in the main text.
